# Complete genome sequence analysis of the peanut pathogen *Ralstonia solanacearum* strain Rs-P.362200

**DOI:** 10.1186/s12866-021-02157-7

**Published:** 2021-04-19

**Authors:** Kun Chen, Lihui Wang, Hua Chen, Chong Zhang, Shanshan Wang, Panpan Chu, Shaokang Li, Huiwen Fu, Tao Sun, Menghan Liu, Qiang Yang, Huasong Zou, Weijian Zhuang

**Affiliations:** 1 College of Plant Protection, Fujian Agriculture and Forestry University, Fuzhou, 350002 Fujian China; 2 College of Agronomy, Fujian Agriculture and Forestry University, Fuzhou, 350002 Fujian China

**Keywords:** *Ralstonia solanacearum*, Peanut, Genome, Effector, Pathogenicity island, Prophage

## Abstract

**Background:**

Bacterial wilt caused by *Ralstonia solanacearum* species complex is an important soil-borne disease worldwide that affects more than 450 plant species, including peanut, leading to great yield and quality losses. However, there are no effective measures to control bacterial wilt. The reason is the lack of research on the pathogenic mechanism of bacterial wilt.

**Results:**

Here, we report the complete genome of a toxic *Ralstonia solanacearum* species complex strain, Rs-P.362200, a peanut pathogen, with a total genome size of 5.86 Mb, encoding 5056 genes and the average G + C content of 67%. Among the coding genes, 75 type III effector proteins and 12 pseudogenes were predicted. Phylogenetic analysis of 41 strains including Rs-P.362200 shows that genetic distance mainly depended on geographic origins then phylotypes and host species, which associated with the complexity of the strain. The distribution and numbers of effectors and other virulence factors changed among different strains. Comparative genomic analysis showed that 29 families of 113 genes were unique to this strain compared with the other four pathogenic strains. Through the analysis of specific genes, two homologous genes (gene ID: 2_657 and 3_83), encoding virulence protein (such as RipP1) may be associated with the host range of the Rs-P.362200 strain. It was found that the bacteria contained 30 pathogenicity islands and 6 prophages containing 378 genes, 7 effectors and 363 genes, 8 effectors, respectively, which may be related to the mechanism of horizontal gene transfer and pathogenicity evaluation. Although the hosts of HA4–1 and Rs-P.362200 strains are the same, they have specific genes to their own genomes. The number of genomic islands and prophages in HA4–1 genome is more than that in Rs-P.36220, indicating a rapid change of the bacterial wilt pathogens.

**Conclusion:**

The complete genome sequence analysis of peanut bacterial wilt pathogen enhanced the information of *R. solanacearum* genome. This research lays a theoretical foundation for future research on the interaction between *Ralstonia solanacearum* and peanut.

**Supplementary Information:**

The online version contains supplementary material available at 10.1186/s12866-021-02157-7.

## Background

*Ralstonia solanacearum* (*R. solanacearum*) is a Gram-negative bacterium, Burkolderiaceae (beta-proteobacteria) with a cell length of 0.5–1.5 μm [1]. *R.solanacearum* is also considered to be *Ralstonia solanacearum* species complex (RSSC) due to the diversity of its genetic group. RSSC can survive in the soil for a long time, and once it can break through the plant defense line, it will enter the vascular bundle and multiply and cause the plant to die, thus returning to the soil again to prepare for the next transmission [2]. In the environment of laboratory aseptic water, *R. solanacearum* can survive for more than 4 years without weakening its pathogenicity, and it can survive for several years without any nutrients [3]. This phenomenon is extremely rare at present. It can be seen that the viability of *R. solanacearum* is very strong. It may also be one of the reasons why RSSC can spread widely in the world*.* RSSC can be divided into four phylotypes corresponding to geographical locations: Asian (phylotype I), American (phylotype II), African (phylotype III) and Indonesian (phylotype IV) [4]. Each phylotype can be subdivided into different sequevars, which may include different strains showing similar pathogenicity or a similar geographic origin [5]. In 2016, after Prior et al. added the relevant data of proteome and metabolic group to the original classification, RSSC were divided into three species: the first species (composed of phylotype I and phylotype III), the second species (composed of phylotype IIA and phylotype IIB), and the third species phylotype IV [6]. At present, this classification method is widely used by most researchers.

RSSC has been rated as the second most important plant pathogenic microorganism in the world, and it has also become a model bacteria for the study of plant-pathogenic microorganism interaction [7]. Bacterial wilt caused by RSSC is one of the most extensive bacterial diseases in the world and can infect more than 450 species of plants in 54 families [8, 9]. Its hosts include not only dicotyledonous herbs such as *Solanaceae* and *Leguminosae* but also dicotyledonous woody plants such as mulberry, eucalyptus and *Casuarina equisetifolia* and monocotyledonous plants such as banana and ginger [10]. Bacterial wilt is an important disease that restricts peanut production in China and many Southeast Asian countries, and ranks first among several bacterial diseases of peanuts [11]. Bacterial wilt disease caused by the *R. solanacearum* Rs-P.362200 strain is a devastating disease in Chinese peanut production that can cause yield losses of up to 50–100% [12, 13].

The pathogenicity of *R. solanacearum* is closely related to its virulence factors. In a nutritious environment, *R. solanacearum* synthesizes extracellular polysaccharides (EPS) to block vascular bundles and hinder water transport, resulting in the death of host plants [14]. In addition to EPS, *R. solanacearum* exhibits many other virulence factors, such as effectors, type 4 fimbriae and polycarboxylate siderophore staphyloferrin B [15, 16]. Type III effector proteins (T3Es) account for a considerable fraction of the many virulence factors, and current research on these proteins is more extensive than that on other virulence factors [17]. *R. solanacearum* uses syringe-like type III secretion system to inject T3Es into plant cells, interfering with the life activities of the host. Some T3Es of *R. solanacearum* play a decisive role in the pathogenic process and are therefore referred to as toxic proteins. A few T3Es can be recognized by plant resistance gene products and stimulate resistant plants to produce a hypersensitive response (HR); these proteins are therefore referred to as avirulent proteins (Avr) [18].

The genomes of microorganisms are relatively small, and with the development of modern sequencing technology, more and more microbial genomes have been sequenced [19, 20]. The sequencing of the whole genome of *R. solanacearum* could provide a theoretical basis for the study of its pathogenic mechanism and gene regulatory network. Since the sequencing of the GMI1000 strain in 2002 [21], an increasing number of strains have been sequenced. To date, the genome assemblies and annotations of 164 *R. solanacearum* strains have been released in the NCBI database (https://www.ncbi.nlm.nih.gov/genome/browse/#!/prokaryotes/490/). HA4–1 is the first strain of *R. solanacearum* isolated from peanut and sequenced in 2019 [17]. Although increasing numbers of strains have been sequenced, more genomic sequences are still needed to fully analyze the species. Strains from different regions and different host sources vary greatly in their host range, pathogenicity, biotype, serotype and other bacteriological characteristics [22].

In the present study, we sequenced the whole genome of the Rs-P.362200 strain. The host-specific candidate genes and the evolutionary relationships of the strain were determined via comparative genomics and evolutionary analysis.

## Results

### Genome sequencing, assembly and annotations

**Fig. 1:**
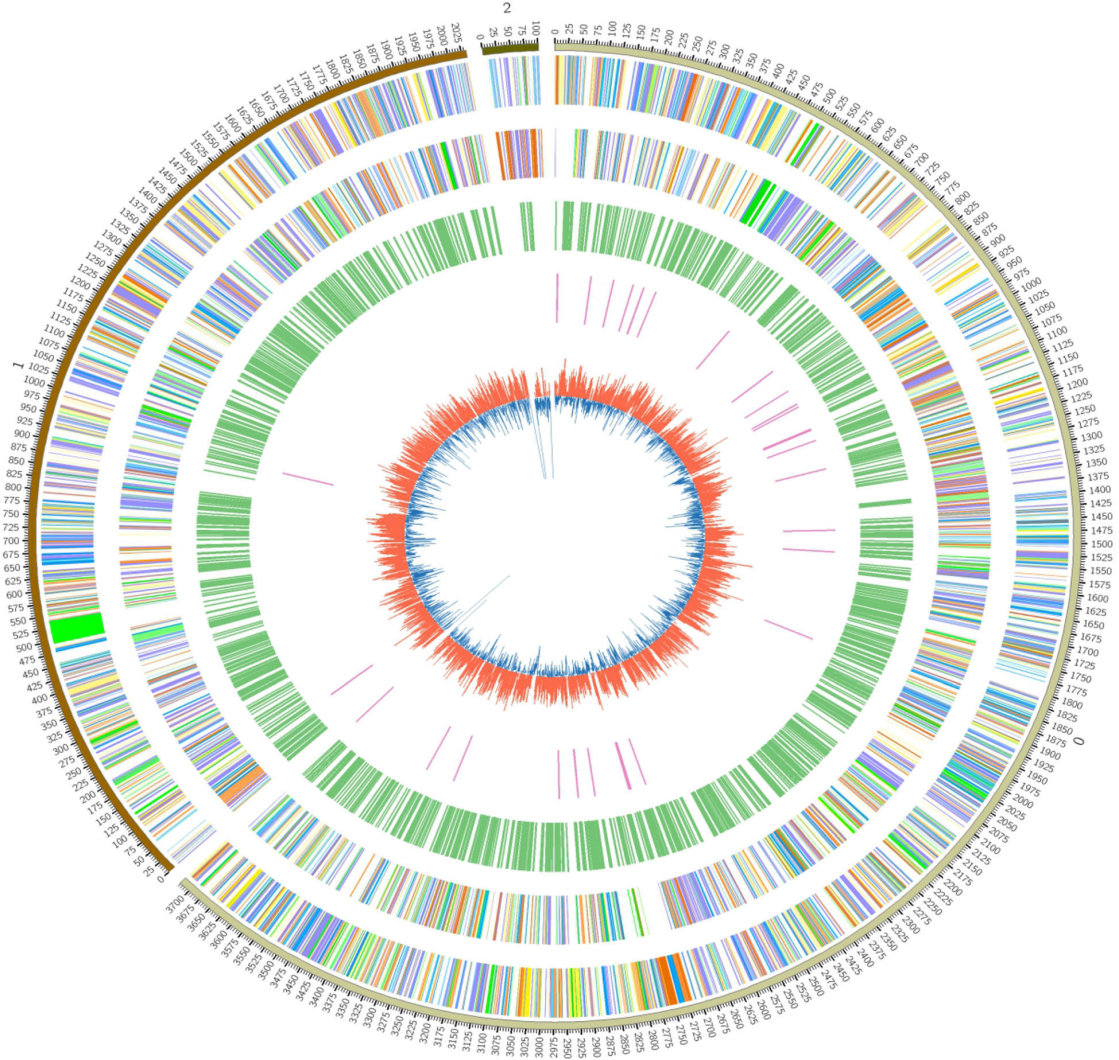
The circular maps of the RS-P362200 genome. The outermost circle indicates the size of the genome (0, 1, and 2 indicate the chromosome, the big plasmid and the small one, respectively, each scale is 0.1 Mb); the second and third circles are genes on the positive and negative strands of the genome, respectively. Different colors represent different COG functional classifications (for the detailed explanations of functional groups of each color, see the Fig. 2). The fourth circle is the repeat sequence; the fifth circle is the tRNA; the innermost layer is the GC content. The red bars of this layer indicates that the GC content in this area is higher than the average GC content of the genome. The blue bars indicates that the GC content in this region is lower than the average GC level of the genome

To understand the interaction mechanism of Rs-P.362200 with peanut from the pathogen perspective, single-molecule real-time sequencing (SMRT) on the PacBio RS II platform was used to sequence the genome of Rs-P.362200. A total of 1.09 Gb of clean data were generated that covered 186 folds of the whole genome size. By following the MinHash Alignment Process (MHAP) [23, 24] workflow, the clean data from the PacBio RS II platform were assembled into three scaffolds (corresponding to 1 chromosome, 1 megaplasmid and 1 small plasmid) of approximately 3.72 Mb, 2.03 Mb, and 101 kb, respectively (Table 1, Fig. 1). The sequences coverage depth of these scaffolds was at least 100X throughout the genome with average coverage from 130X ~ 215X. The mean confidence of these scaffolds was close to QV50 (Supplementary 1), thereby we got a complete genome sequences.

**Table 1:** Results of genome assembly

Label	Size (bp)
Chromosome	3,721,710
Megaplasmid	2,034,020
Small Plasmid	101,780
Total	5,857,510

The average G + C content of the genome was 67%. The general characteristics of the Rs-P.362200 genome are listed in Table 2. Overall, 5056 coding genes were predicted in this genome with 3342, 1608 and 106 genes located in the chromosome, the megaplasmid and the small plasmid, respectively. Different strategies were used to predict noncoding RNA. The Rs-P.362200 genome contained 408 rRNAs, 36 tRNAs and 5 microRNAs. In this strain, 12 pseudogenes were identified. By using the predicted genome information and drawing a circular genome map, we can more clearly explore the distributions of genes between genome components (Fig. 1).

**Table 2:** Genomic characteristics of the Ralstonia solanacearum Rs-P.362200 strain

Type	Chromosome	Megaplasmid	Small plasmid	Total
Coding gene	3342	1608	106	5056
miRNA	3	2	0	5
rRNA	306	102	0	408
tRNA	34	2	0	36
Pseudogene	3	6	3	12

**Fig. 2:**
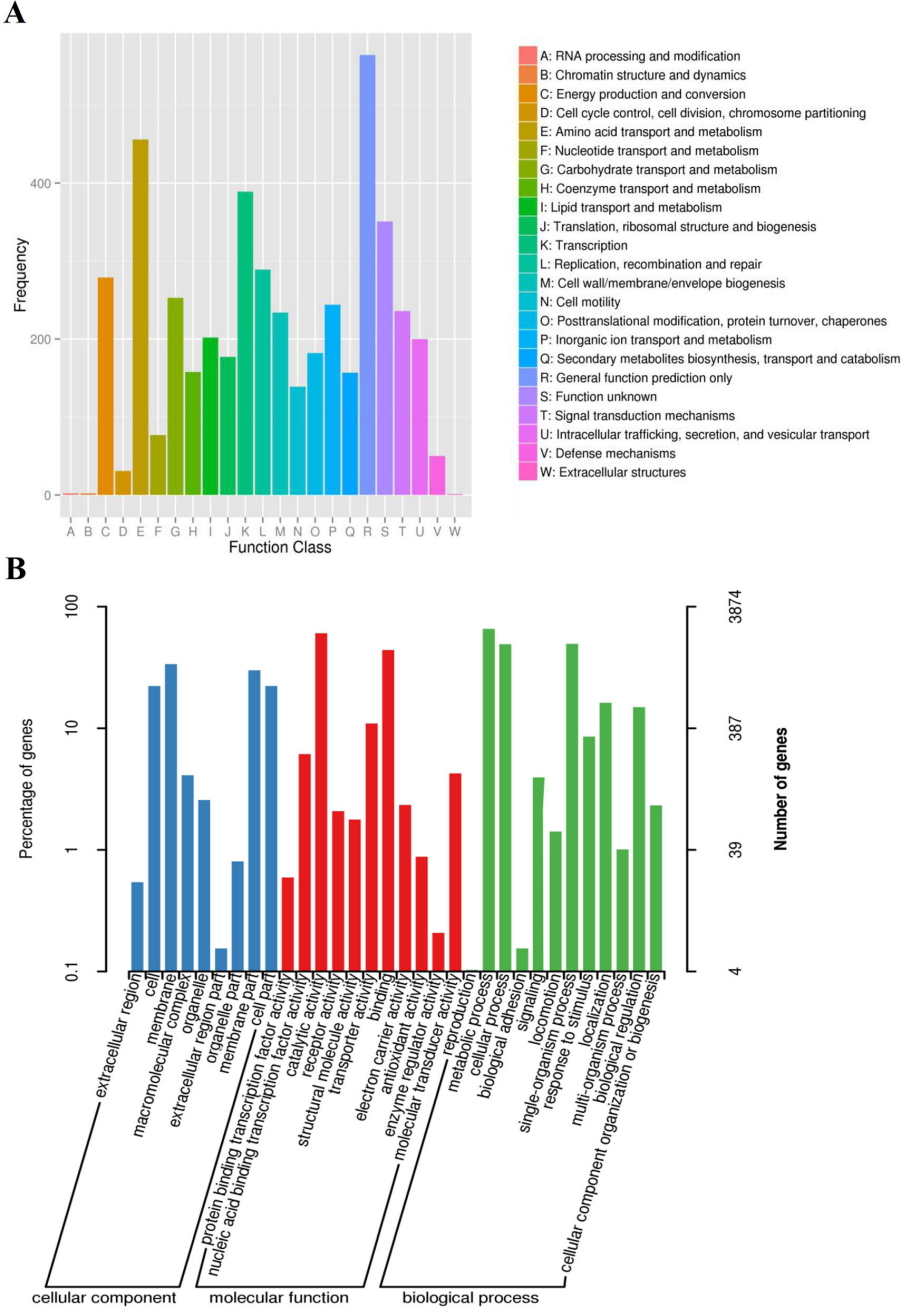
Functional classification analysis in Rs-P.362200 genome. **a** COG classification statistics: Letters along the abscissa is the content of functional classification of COG, and the ordinate is the number of genes. **b** GO classification statistics: The abscissa is the name of each GO classification, the left of the ordinate is the percentage of the number of genes, and the right is the number of genes

**Fig. 3:**
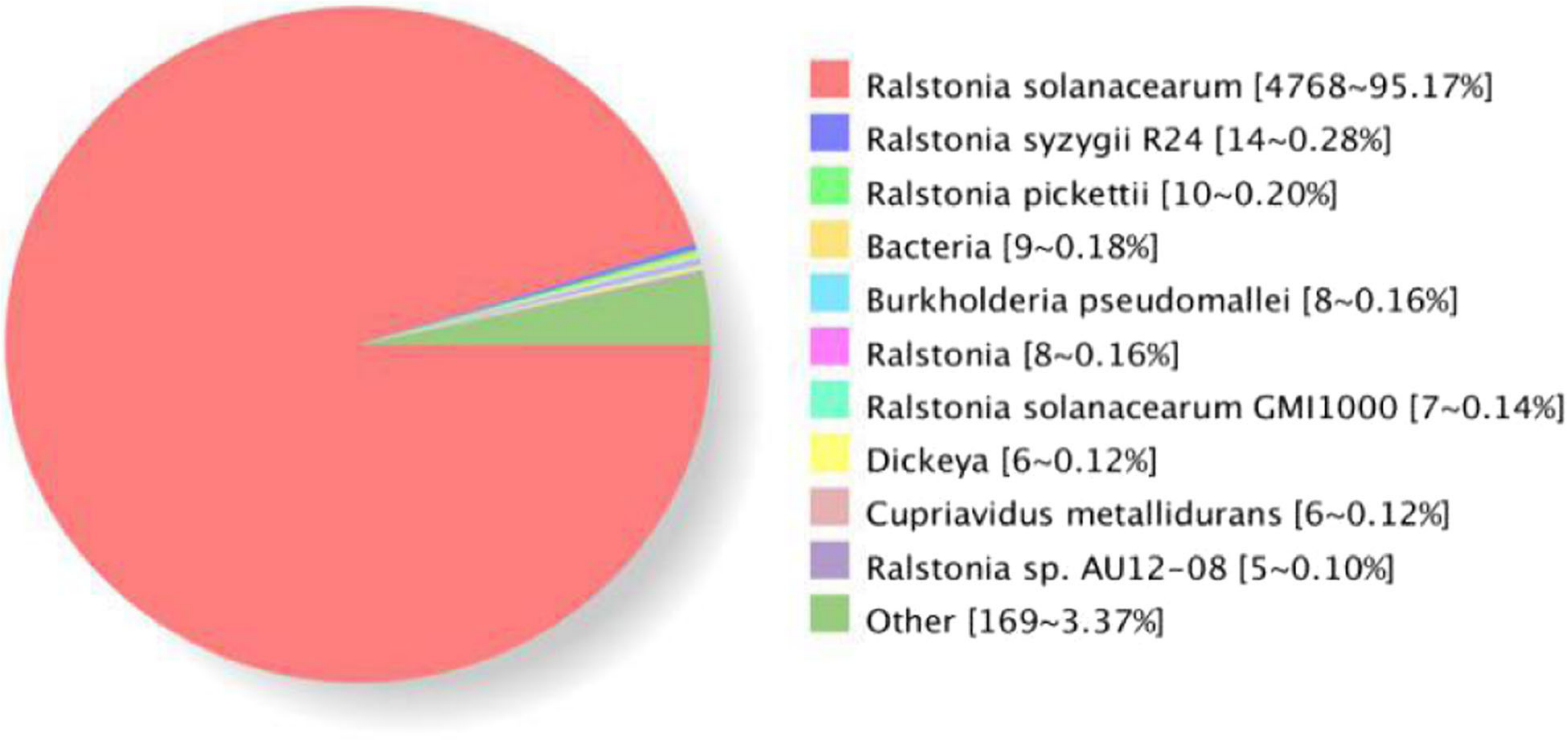
NR species distribution statistics. This map reflects the distribution of different homologous species genome annotated by NR. Different colors represent different homologous species

The predicted gene sequences were functionally annotated by using BLAST and COG, GO, and NR databases. COG and GO functional classification analyses were performed (Fig. 2). The NR species distribution statistics revealed that 95.17% of the genes belonged to the *R. solanacearum* family, which demonstrated that the strains that we sequenced were of very high quality (Fig. 3).

### Identification and comparative analysis of the virulence factor of Rs-P.362200

Type III effectors (T3Es) are key to the pathogenicity of *R. solanacearum*. Searched on the T3Es protein database [25], 75 effectors were found in the Rs-P.362200 genome and different strains contain varied number of effectors. Compared with 6 reported virulent strains quite diversity presented in the effectors similarity and/or coverage and 20 effectors showed less diversity between Rs-P.362200 and HA4–1 genomes (Table 3). RipAC, RipE2, RipJ, and RipT exhibited two copies in Rs-P.362200 genome (Table 3). However, RipP1 presented three copies in the genome, which were located on the chromosome, the large plasmid and the small plasmid. The effectors that were absent in the reference genome were RipAH, RipE2 (geneID: 3_27), and RipP1 (geneID: 2_657, 3_83) (Table 3). The three effectors that were present in Rs-P.362200 and absent in the reference genomes were subjected to BLAST searches in the NCBI database, and it was found that other genomes contain homologous genes. It is worth noting that according to T3Es and NCBI databases, RipP1 (geneID: 2_657, 3_83) exists only in RSCM and HA4–1 genomes (Supplementary 2).

**Table 3:** Comparison analysis of type III effector proteins genes (Coverage%/Identity%) in Rs-P.362200 and other strains

Effector name	Rs-P.362200 gene ID	GMI1000	YC45	CMR15	PSI07	Po82	HA4–1
RipA1	1_3262	95/99	100/98	absent	absent	absent	100/100
RipA2	2_1476	100/100	100/100	100/93	100/79	97/78	100/100
RipA3	2_596	96/98	90/54	100/90	96/82	100/69	96/99
RipA4	2_597	100/99	96/99	100/89	100/80	90/53	100/100
RipA5	2_793	99/97	99/97	absent	100/79	97/55	99/99
RipAB	2_627	100/99	100/99	99/93	100/77	100/70	100/100
RipAC	2_625	97/100	absent	97/46	99/69	93/52	absent
	2_626	99/99	absent	99/52	99/80	99/66	absent
RipAD	2_1349	100/98	100/99	100/80	100/74	98/69	100/100
RipAE	2_617	100/99	100/100	100/97	100/96	100/97	100/100
RipAF1	2_571	100/98	absent	100/87	absent	100/58	100/100
RipAH	1_1184	absent	absent	absent	absent	absent	absent
RipAJ	1_3299	100/99	100/100	100/73	100/69	100/71	100/100
RipAK	1_3050	100/100	100/99	absent	absent	absent	100/100
RipAL	2_480	absent	absent	99/83	100/99	96/81	100/100
RipAM	1_2241	100/100	100/100	100/94	100/83	89/74	100/100
RipAN	2_595	97/99	100/99	97/85	92/74	97/70	96/99
RipAO	2_630	100/97	100/98	100/83	100/54	absent	100/100
RipAP	2_982	absent	100/99	100/84	absent	100/79	100/100
RipAQ	2_621	100/99	100/100	100/98	100/93	100/92	100/100
RipAR	2_1003	100/98	100/99	100/83	95/72	99/65	95/57
RipAS	2_1121	89/99	100/99	100/89	absent	97/52	100/100
RipAT	2_1125	74/98	100/99	99/64	74/71	99/63	74/99
RipAU	2_1202	100/97	100/98	100/82	99/70	99/70	100/100
RipAV	2_488	100/98	100/98	100/88	absent	100/87	100/100
RipAW	2_1218	82/97	82/98	82/85	100/66	100/63	82/99
RipAX2	2_329	89/100	absent	absent	absent	absent	88/100
RipAY	2_791	83/95	100/99	100/83	95/67	100/60	82/99
RipB	1_1817	100/100	100/99	98/80	100/79	100/72	100/100
RipBC	1_2236	100/100	100/100	100/99	100/98	100/93	100/100
RipBF	2_1512	100/99	absent	100/96	absent	100/96	100/100
RipBM	2_1577	absent	absent	100/90	100/94	absent	100/100
RipC1	2_1006	100/98	100/98	absent	95/96	96/70	100/100
RipC2	2_94	84/91	100/98	100/67	100/83	100/63	100/100
RipE1	1_2140	100/99	absent	100/87	100/79	100/85	100/100
RipE2	1_1224	absent	absent	absent	78/97	100/89	100/100
	3_27	absent	absent	absent	absent	absent	absent
RipF1	2_640	85/96	86/95	85/94	86/85	85/87	85/94
RipG1	2_649	100/99	100/99	absent	92/53	absent	100/100
RipG2	2_430	100/98	100/96	100/81	100/64	100/73	94/99
RipG3	2_1452	100/98	100/98	100/86	100/71	100/59	100/100
RipG4	1_418	87/99	100/99	100/78	100/51	100/58	86/99
RipG5	1–419	81/99	81/99	80/89	81/74	81/65	100/100
RipG7	1_645	98/69	98/69	98/68	99/73	99/65	100/100
RipH1	1_571	100/97	100/97	100/82	98/65	98/39	100/99
RipH2	2_1579	92/97	100/97	100/82	98/55	98/49	100/100
RipH3	2_1525	100/99	100/99	100/86	98/71	99/79	100/100
RipI	1_2027	100/100	100/100	100/87	100/93	100/82	100/100
RipJ	2_653	98/71	absent	94/26	absent	100/72	100/100
	2_679	97/67	absent	absent	absent	97/72	97/71
RipL	2_1560	100/99	100/97	99/81	absent	100/61	100/100
RipM	1_483	100/99	100/98	100/89	99/80	absent	100/100
RipN	2_905	100/98	100/99	100/89	98/73	100/73	100/100
RipO1	2_116	100/99	absent	100/91	absent	96/86	100/100
RipP1	1_1183	100/95	absent	absent	absent	100/95	100/100
	2_657	absent	absent	absent	absent	absent	100/100
	3_83	absent	absent	absent	absent	absent	100/100
RipP2	1_3098	100/100	100/100	100/96	absent	100/84	100/100
RipQ	2_1046	70/99	70/99	70/76	absent	100/71	69/99
RipR	2_1050	100/100	100/100	100/91	100/82	100/80	100/100
RipS1	1_2108	100/99	100/99	100/91	100/75	100/89	99/70
RipS2	2_1105	98/99	91/50	98/91	99/78	98/75	100/99
RipS3	2_699	100/98	100/98	100/92	100/77	100/76	100/100
RipS4	1_454	98/98	99/99	99/83	91/62	97/72	92/63
RipS5	2_90	100/99	100/99	100/85	100/76	100/70	100/100
RipS6	1_3270	89/99	89/99	94/49	93/48	91/49	89/99
RipS7	1_3142	100/99	100/100	100/97	100/92	100/92	100/100
RipS8	1_457	79/63	100/98	78/61	100/90	78/63	100/100
RipT	1_1155	71/100	absent	absent	99/89	absent	100/100
	1_1221	71/87	absent	absent	100/94	absent	99/90
RipTAL	1_432	100/99	100/99	absent	64/75	absent	100/100
RipTPS	2_487	100/99	100/99	100/96	94/76	80/44	100/100
RipU	2_979	absent	100/99	absent	100/77	100/78	100/100
RipV1	1_653	61/97	61/97	58/85	54/71	53/63	100/100
RipW	1_2687	100/99	100/99	100/90	100/84	100/78	100/100
RipX	2_628	100/95	100/95	100/77	100/74	99/63	100/100
RipY	1_1806	99/62	99/62	99/61	99/61	99/60	100/100
RipZ	2_612	100/99	100/99	100/96	100/88	100/86	100/100
RS-T3E-Hyp12	2_1563	100/99	100/100	100/94	100/90	100/81	100/100
RS-T3E-Hyp6	1_1198	absent	absent	71/90	absent	absent	72/99
RS-T3E-Hyp8	2_216	100/100	100/99	100/99	100/97	100/94	100/100
Total	75	71	61	65	61	66	77

Type three secretion system can inject effector proteins into plants, making them susceptible to diseases. We compared the structural gene clusters of type three secretion system between Rs-P.362200 and HA4–1 (Supplementary 3). Except PopC and hrcC, their genes are almost completely similar. At the same time, other virulence factors were compared, and excepting PehR, there was no other difference in virulence factors between the two strains (Supplementary 3).

### Phylogenetic analysis

**Fig. 4:**
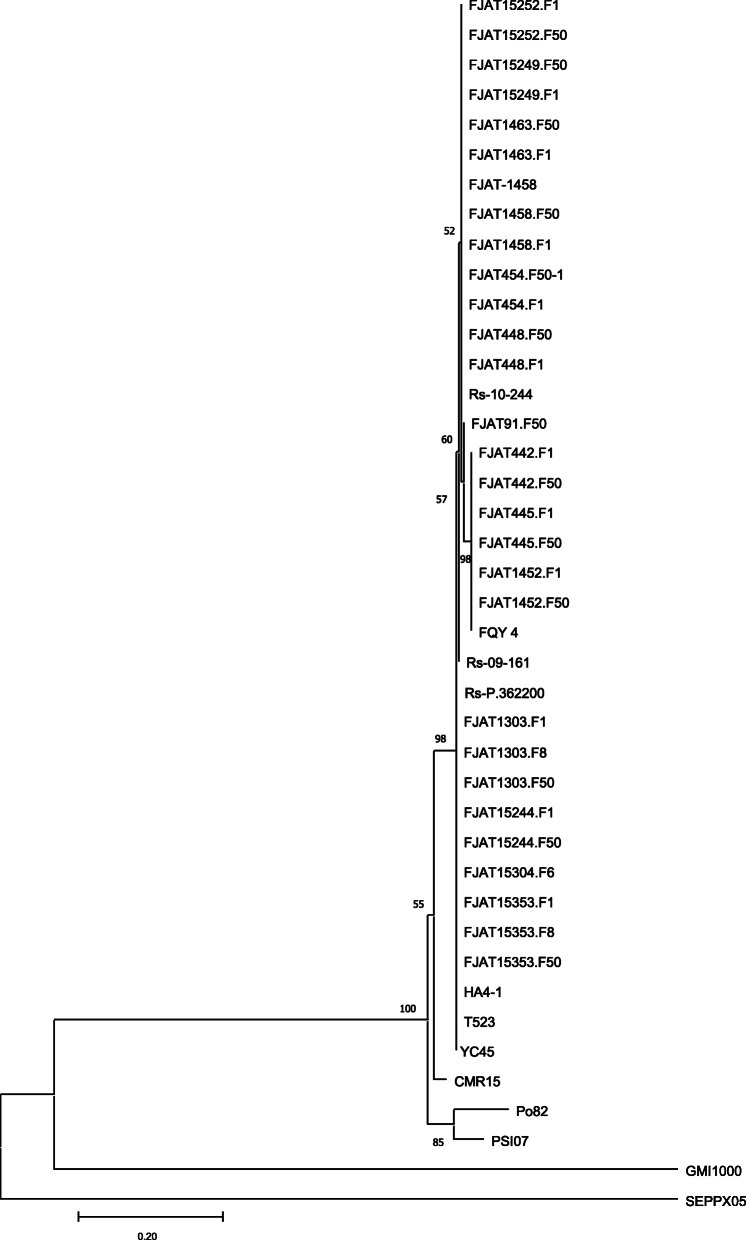
Genetic relationship between Rs-P.362200 and other virulent strains. The evolutionary relationship was inferred using the Neighbor-Joining method. Phylogenetic tree based on the comparison of endoglucanase gene sequence from *R. solanacearum* strains. The percentage of replicate trees in which the associated taxa clustered together in the bootstrap test (1000 replicates) are shown next to the branches. The bottom 0.2 scale represents two nucleotide change per 100-nucleotide position

We downloaded the entire genome sequences of 40 *R. solanacearum* strains that have been sequenced from NCBI. Among these strains, GMI1000 and YC45 belong to phylotype I, the Po82 strain belongs to phylotype II, the CMR15 strain belongs to phylotype III, and the PSI07 strain belongs to phylotype IV. The rest of the strains were isolated from different regions and hosts in China and belong to phylotype I, and the strain information used to construct the phylogenetic tree is shown in Supplementary 4. Phylogenetic trees were drawn based on the similarity of endoglucanase gene sequence from the above strains and Rs-P.362200 (Fig. 4). Phylogenic analysis places SEPPX05 and GMI1000, belonging phylotype I, as outgroup strains which diversed far from the other strains. Po82, PSI07 and CMR15 representing of respective phylotypes of II, IV and III, were also placed far from the other phylotype I strain, isolated from Asia China and India (Supplementary 4). Aparently, phylotype I diversified greatly as depending to the origins and infected plants.

### Comparative genomic analyses

Collinear genes comparisons of Rs-P.362200 with six other reference *R. solanacearum* genomes were performed, which indicated that inversions and translocations are main events for genomes diversity among RSSC (Supplementary 5), with only one translocation between chromosomes of Rs-P.362200 and AH4–1 and two inversons in the megaplasmid. The percentage of collinear genes between Rs-P.362200 and HA4–1 was highest, which incoincided with the evolutionary relationships between the previous strains (Po82, CMR15, PSI07) (Fig. 4, Table 4).

**Table 4:** Comparison of collinearity between Rs-P.362200 and other strains of Ralstonia solanacearum

Strain name	Collinear with Rs-P.362200 (%)
HA4–1	88.75
GMI1000	80.68
CMR15	74.89
YC45	72.74
Po82	70.4
PSI07	69.66

**Fig. 5:**
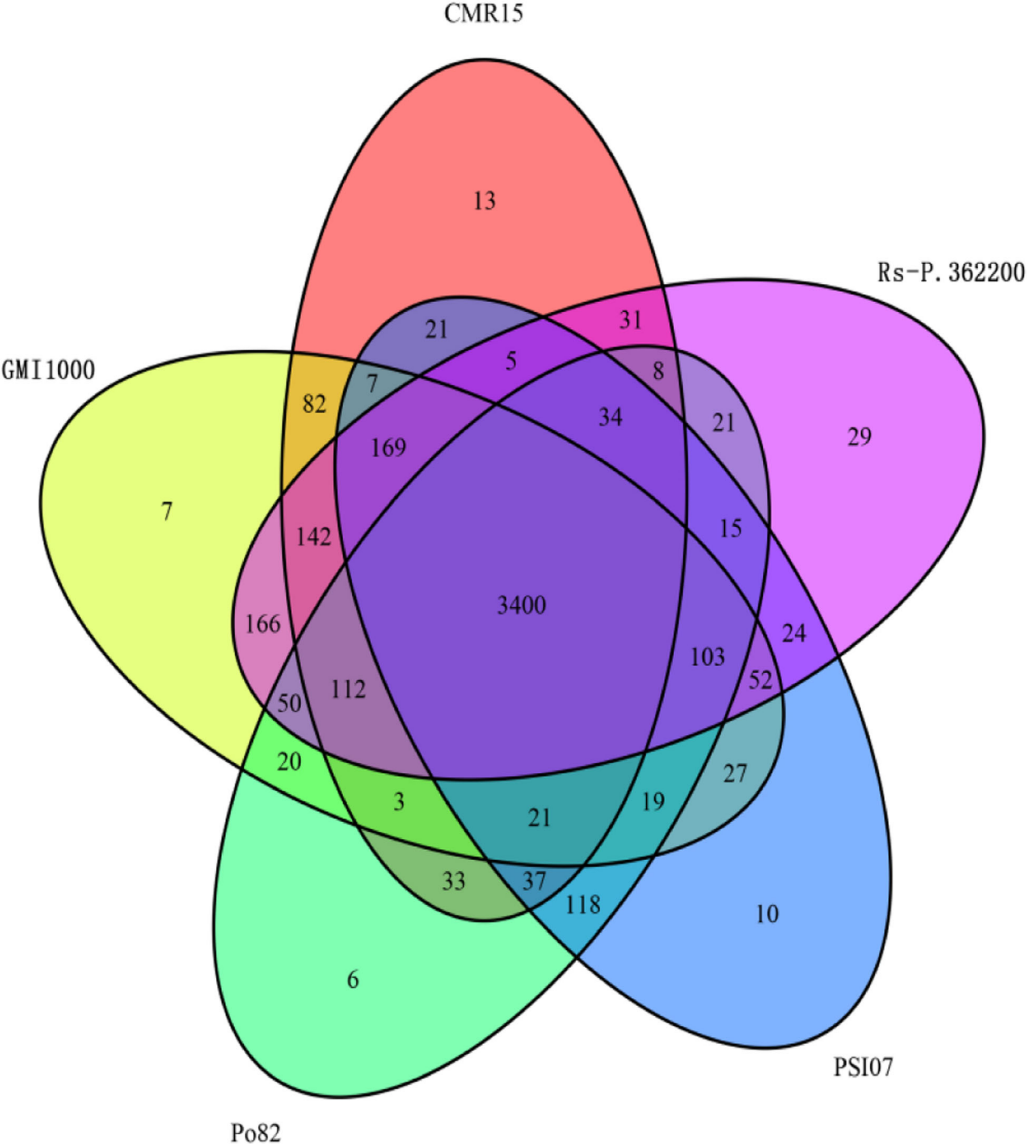
Venn diagram showing the orthologous genes shared between or distinct to the indicated *R. solanacearum* strains Rs-P.362200, GMI1000, CMR15, Po82 and PSI07. There are 3400 common protein-coding gene clusters for these five types of strains and Rs-P.362200 contains more specific gene families

Genomic comparison of the Rs-P.362200 strain with the 4 other types of strains were carried out using the genomic protein sequences, and the unique gene families of this strain were identified. Gene family analysis showed that there were 4812 genes clusters in the genome of the Rs-P.362200 strain, which could be classified into 4361 gene families, among which 29 gene families (including 113 genes) were unique to Rs-P.362200 (Fig. 5, Table 5, Supplementary 6). Most of the 113 genes unique to this strain encode hypothetical proteins, transposases, putative membrane proteins and phage integrases. Enrichment of 113 unique genes indicates that Rs-P.362200 unique genes are involved in biological processes and molecular functions in GO (Supplementary 7). Two homologous genes (gene ID: 2_657 and 3_83) encode an avirulence protein (RipP1). These genes and effector proteins may associated with the host range of the Rs-P.362200 strain. Of the genomes that have been sequenced so far, only HA4–1 is isolated from peanuts and makes potatoes susceptible to disease. At present, the pathogenicity of Rs-P.362200 strain to other plants has not been reported. However, we have used the Rs-P.362200 isolated from peanut to inoculate tobacco without any infection. We compared the genomic information of HA4–1 and Rs-P.362200, and the number of genomic islands and Prophages of Rs-P.362200 genome was less than that of HA4–1(Supplementary 8–1). Comparing HA4–1 and Rs-P.362200 genomes, there are 147 gene families unique to HA4–1 genome and 151 gene families unique to Rs-P.362200. Enriching the unique gene family showed that the unique gene of HA4–1 participated in the biological process and molecular function in GO, while the unique gene of Rs-P.362200 participated in the biological process in GO (Supplementary 9).

**Table 5:** Gene family classification statistics

Species name	Total gene number	Gene cluster number	Total family number	Unique gene
				family number
CMR15	4749	4361	4118	13
GMI1000	4833	4663	4380	7
PSI07	4664	4397	4062	10
Po82	4526	4279	4000	6
Rs-P.362200	5056	4812	4361	29

### Horizontal gene transfer is extensively observed in the Rs-P.362200 genome

Many studies have shown that horizontal gene transfer (HGT) is the main driving force for the evolution of prokaryotes, affecting all aspects of prokaryotes [26, 27]. Pathogenicity islands (PAIs) are related to the pathogenesis of bacteria, and some pathogenic genomic islands can cause horizontal gene transfer in closely related species. Through PAI analysis, it was found that 378 genes in the Rs-P.362200 strain were distributed in 30 PAIs (Supplementary 8–2). Most genes in the PAIs were hypothetical protein-encoding genes, and some were type III effector protein genes, such as RipAX2, RipB, RipT, RipP1 (geneID: 1_1183), RipAH, RS-T3E-Hyp6 and RipE2 (geneID: 1_1224).

Horizontal gene transfer in prokaryotes is mainly achieved through three mechanisms: transformation, conjugation, and transduction [28]. Transduction is carried out by bacteriophages, which can be integrated into bacterial chromosomes in the form of prophages and remain latent for a long time. In this study, 363 genes in 6 prophages were identified (Supplementary 8–3). RipE2 (geneID: 3_27), RipAK, RipP2, RipT, RipP1 (geneID: 1_1183), RipAH, RS-T3E-Hyp6 and RipE2 (geneID: 1_1224) were distributed in prophage sequences. Interestingly, RipP1 (geneID: 1_1183), RipAH, RS-T3E-Hyp6 and RipE2 (geneID: 1_1224) were located in genomic islands, and prophages. These genes, co-existing in genomic islands and prophages, are found in strains from different sources (Supplementary 2). May be this pathogenic genes obtained from other bacteria through horizontal gene transfer.

## Discussion

RSSC is one of the ten most harmful plant pathogens in the world and can cause the withering of many important crops. Understanding of the whole-genome characteristics of RSSC is helpful for studying the pathogenic mechanism of *R. solanacearum* at the gene level to provide a theoretical basis or strategies for the effective control of bacterial wilt. Since the whole-genome sequencing of the first *R. solanacearum* strain, GMI1000, in 2002 [12], molecular biology and microbial whole-genome sequencing techniques have been considerably improved, and an increasing number of strains have been sequenced. Although *R. solanacearum* was found on peanuts in 1930s, it was not until 2019 that Tan first released the strains isolated from peanuts [17]. Conquering peanut bacterial wilt is a worldwide problem, but there are few studies on the pathogenic mechanism of peanut bacterial wilt. According to the current research, HA4–1 can make peanut and potato susceptible. However, whether Rs-P.362200 will make other plants such as potato susceptible to disease remains to be explored further, yet it cannot infect tobacco plants. The comparative genomic analysis of Rs-P.362200 and other reference genomes revealed the diversity of Type III effector proteins and host-specific candidate genes. The comparison of genomic information between HA4–1 and Rs-P.362200 shows that the number of genomic islands and prophages in HA4–1 genome is more than that in Rs-P.362200. Maybe it has something to do with the host range of the strain, and more strains isolated from peanuts need to be sequenced to better explain this problem.

Many Type III effector proteins have been identified as virulence factors or avirulent proteins in *R. solanacearum*. Macho and other authors have shown that RipD, RipP2, RipAC, RipY, RipA1, RipA2 and RipD can improve the adaptability of *R. solanacearum* in the host. RipW, RipAR and eight proteins of the RipG family can form E3 ubiquitin ligases in the host, which ubiquitinate host defense-related proteins, thereby disrupting the host defense response [29, 30]. Yuying et al. showed that RipAY synthesized g-glutamylcyclotransferase in the host to reduce the activity of glutathione to inhibit the plant immune response [31]. Some effectors play the role of avirulence proteins, which can induce an immune response in the host plant and make the host resistant to disease, as observed for RipAA, RipP1, RipAX1, and RipB [25, 32–34]. In our study, according to the effector protein database and gene functional annotation, 75 type III effector proteins were identified in the Rs-P.362200 genome. Two homologous genes of RipP1 (geneID: 2_657 and 3_83) only exist in RSCM and HA4–1 genomes. The geographical location of strain HA4–1 and RSCM is in Asia, the host of HA4–1 is peanut and the host of RSCM is *Cucurbita maxima*. It is possible that these two genes are the key virulence factors in the pathogenic process of some special hosts. At present, the homologous gene of RipE2 (3_27) is only found in three strains (UA-1612, UA-1611 and IBSBF1503) isolated from South America. The diversity of *R. solanacearum* T3Es may determine the host range and pathogenicity of *R. solanacearum*.

Based on the comparative genomic analysis of Rs-P.362200 and 5 other reference genomes for *R. solanacearum*, it was found that there were 113 genes unique to the Rs-P.362200 genome*.* Two of these specific genes belong to the RipP1 (geneID: 2_657 and 3_83) gene family of pathogenic factors of the *R. solanacearum*, which may be related to host specificity. Their function can only be identified by constructing gene mutants and performing corresponding phenotypic analysis in the later stage. Although the hosts of HA4–1 and Rs-P.362200 strains are the same, they have specific genes belonging to their own genomes (Supplementary 9). The genetic diversity of *R. solanacearum* strains may be the reason for the wide host range and difficulty in control of bacterial wilt at present.

Horizontal gene transfer can enhance the adaptability of bacteria to the environments, and genome islands and prophages are the most important mobile elements in HGT [35]. The coding regions of genome islands usually contains large numbers of virulence gene clusters which encode the virulence factors of many pathogenic bacteria [26]. The nucleic acids of mild bacteriophages, i.e. prophage sequences may allow some bacteria to acquire antibiotic resistance, enhance bacterial adaptability to the environments, improve bacterial adhesion or cause the bacteria to become pathogenic [36]. The analysis showed that the strain contained 30 genomic islands and 6 prophages. Interestingly, RipT, RipP1, RipAH, and RipE2 were found in both genomic islands and prophages. These effector factors may be obtained from other bacterial genomes and transferred to other bacterial genomes via horizontal gene transfer. The strains containing these genes may play an important role in the pathogenicity and adaptation of *R. solanacearum* in the environment. Although these phenomena contribute to explaining the wide host range and high pathogenicity of *R. solanacearum*, subsequent experiments are needed to verify their occurrence.

The evolutionary relationships among *R. solanacearum* strains are closely related to their geographical origin [10]. Kangetal clarified the genetic diversity of *R. solanacearum* in the Yangtze River Valley and southern China, and 95 *R. solanacearum* strains from 9 main peanut-producing areas have been identified as belonging to phylotype I (Asian branch type) [37]. Phylogenetic analysis of 41 strains from 4 phylotypes mainly type I strains demonstrated that type I strains from tomatos they can be classified into different groups because of geographical origins, and the diverse types and hosting plants also make the diversity (Fig. 4). The genetic relationship between Rs-P.362200 and other pathogenic strains of tomatoes is similar (Fig. 4; Supplementary 4). It can be inferred that the genetic relationship between strains has little to do with whether the host is the same or not. The results again confirmed that the high diversity of the *R. solanacearum* species complex makes the species with the widest range of hosts.

## Conclusions

In this study, novel complete genome of the peanut bacterial wilt pathogen was sequenced with distinct diversity. Comparative genomic analysis of different phylotypes of strains provides the evidence for the genetic diversity and host specificity. The reason of wide host range and strong adaptability of *R. solanacearum* was further validated from the events of horizontal gene transfer and the diverse strains with the same host of peanut. The evolutionary relationship between *R. solanacearum* strains was indicated to be more related to geographic origins than the host variance. In short, the results provide an important basis for understanding the pathogenic mechanism of peanut bacterial wilt and lays a theoretical foundation for future research on the interaction between *R. solanacearum* and peanut.

## Materials and methods

### Preparation of strains

The RS-p.362200 strain was donated by the Fujian Academy of Agricultural Sciences (isolated from pathogenic plants in main peanut production area at Fuqin city, Fujian Province in China in 2014). Single colonies were selected after 2 days of culture in TTC medium (1 g hydrolyzed casein, 5 g glucose, 10 g peptone, 0.5 g 2,3,5-triphenyltetrazolium chloride, 15 g agar, dissolved in 1 L water, pH 7.4.) at 28 °C. The selected clones were grown in SPA liquid medium (0.5 g KH_2_PO_4_, 20 g sucrose, 0.25 g MgSO_4_, 5 g peptone, dissolved in 1 L water, pH 7.4.) for 12 h at 28 °C, followed by centrifugation at 4000 rpm for 10 min to collect cells. The prepared strains were used for subsequent experiments.

### Genome sequencing and assembly

Genomic DNA was extracted with the TIANamp Bacteria DNA Kit (TIANGEN Beijing). A 20 kb library was constructed from the bacterial genome and sequenced via the single-molecule real-time (SMRT) sequencing method [38, 39] developed by Pacific Biosciences to obtain sequencing data. The assembly software MHAP [23, 24] was used to assemble the filtered subread data.

### Genome structure analysis

RepeatMasker software [40] was used to mask the repetitive sequence of the bacterial genome. The coding genes of the assembled genome were predicted with the software prodigal [41]. The predicted protein sequences were used to identify homologous gene sequences in the NCBI database by using BLASTP [42], after which immature stop codons and frameshift mutations in the gene sequences were then identified by GeneWise [43], and pseudogenes were annotated. IslandPath-DIOMB software [44] was used to predict the pathogenicity islands in the bacterial genome. The software PhiSpy [45] was used to predict the prophages. And the predicted genomic information, such as repeat sequences and GC content, was used to draw the circular genome map with the CIRCOS tool [46].

### Functional annotation of the genome

Gene function annotations were performed based on the NR (Non-Redundant Protein Database) [47], COG (Clusters of Orthologous Groups) [48], and GO (Gene Ontology) [49] databases. Type III effector proteins were predicted by using the T3E database [25]. The clusterProfiler software [50] was used for the enrichment analysis of GO and KEGG.

### Identification of orthologous genes

The protein sequences of *R. solanacearum* RS-P.362200, GMI1000, Po82, CMR15, PSI07 and HA4–1 were classified with OrthoMCL software [51] to identify the specific gene family of the strains. The identification of orthologs among the 6 *R. solanacearum* strains was also performed via OrthoMCL analysis. The protein sequences of putative orthologous groups including only single-copy genes (one-to-one orthologs) that were shared by all *R. solanacearum* strains were aligned using MUSCLE software with the default parameters [52]. Single-copy genes were identified as those for which only one gene per *R. solanacearum* strain was included in the orthologous group. Comparative analysis of orthologs and the copy numbers was performed among RS-p.362200 and the other strains for visualization with InteractiVenn using Custom Perl scripts.

### Construction of phylogenetic tree

The evolution relationship among 41 strains including all four phylotyps was inferred using the Neighbor-Joining method [53]. The optimal tree with the sum of branch length = 2.61573329 is shown. The percentage of replicate trees in which the associated taxa clustered together in the bootstrap test (1000 replicates) are shown next to the branches [54]. The tree is drawn to scale, with branch lengths (next to the branches) in the same units as those of the evolutionary distances used to infer the phylogenetic tree. The evolutionary distances were computed using the Poisson correction method [55] and are in the units of the number of amino acid substitutions per site. The analysis involved amino acid sequences. All ambiguous positions were removed for each sequence pair. There were a total of 498 positions in the final dataset. Evolutionary analyses were conducted in MEGA7 [56].

### Comparative genomic analysis

The Multiple Collinearity Scan Toolkit (MCScanX) was employed to search for collinear genes between RS-p.362200 and the other 5 *R. solanacearum* strains [57]. As a sign of collinearity between genomes, the percentage of collinear genes in each paired strain (RS-p.362200 vs GMI1000, RS-p.362200 vs CMR15, RS-p.362200 vs YC45, RS-p.362200 vs Po82, RS-p.362200 vs PSI07, and RS-p.362200 vs HA4–1) was also analyzed (Supplementary 2).

## Supplementary Information

**Additional file 1: Supplementary 1.** Compare efficiency statistics.

**Additional file 2: Supplementary 2.** The distribution of specific effector proteins in *Ralstonia solanacearum* species complex.

**Additional file 3: Supplementary 3.** Type III secretion system and other virulence factors.

**Additional file 4: Supplementary 4.** The strain information used to construct the phylogenetic tree.

**Additional file 5: Supplementary 5**. The collinear relationship between Rs-P.362200 strain and other strains.

**Additional file 6: Supplementary 6.** Gene analysis related to specific genes in Rs-P.362200.

**Additional file 7: Supplementary 7.** Go classification statistics of specific genes of Rs-P.362200 strain.

**Additional file 8: Supplementary 8-1.** Comparative of HA4-1 and Rs-P.362200 genomic features. **Supplementary 8-2.** Analysis of genes in genomic pathogenicity islands. Supplementary 8-3. Analysis of genes in prophages.

**Additional file 9: Supplementary 9.** Comparative genomic analysis of R. solanacearum strains Rs-P.362200 and HA4-1.

## Data Availability

The pacbio RSII rawdata of RS WGS was deposited in the NCBI SAR database under the accession number PRJNA668065, is available from [https://www.ncbi.nlm.nih.gov/bioproject/?term=PRJNA668065].
