# Supplementary material for: Complete genome sequence analysis of the peanut pathogen Ralstonia solanacearum strain Rs-P.362200
Source: BMC Microbiol. 2021 Apr 19;21:118. doi: 10.1186/s12866-021-02157-7 (PMC8056632; doi:10.1186/s12866-021-02157-7)

**Supplementary 1 Compare efficiency statistics.** Abscissa represents the average coverage of sequencing. The ordinate represents the sequencing accuracy, and the calculation formula is as follows: QV=-10*log10 (p), where p is the sequencing base error rate. For example, the accuracy of QV20 is 99% and the accuracy of QV50 is 99.999%.


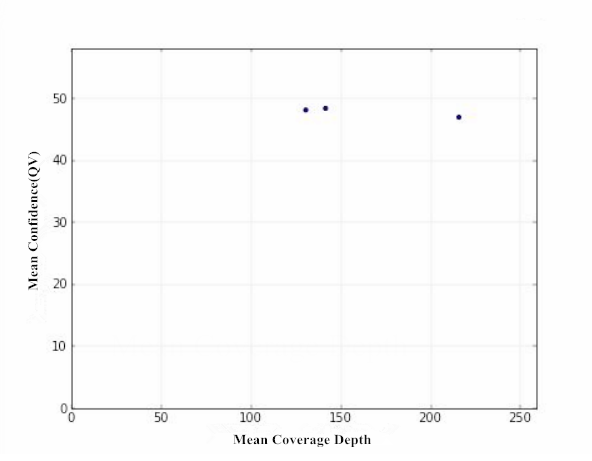

Supplement: Supplementary file 1 — Additional file 1: Supplementary 1. Compare efficiency statistics. [file 12866_2021_2157_MOESM1_ESM.docx]
