# Supplementary material for: Complete genome sequence analysis of the peanut pathogen Ralstonia solanacearum strain Rs-P.362200
Source: BMC Microbiol. 2021 Apr 19;21:118. doi: 10.1186/s12866-021-02157-7 (PMC8056632; doi:10.1186/s12866-021-02157-7)
Supplement: Supplementary file 5 — Additional file 5: Supplementary 5. The collinear relationship between Rs-P.362200 strain and other strains. [file 12866_2021_2157_MOESM5_ESM.docx]

**Supplementary 5 The collinear relationship between Rs-P.362200 strain and other strains.** The genome location coordinates of the reference near-source species are measured on the left, and the assembled genome location coordinates are on the right. The lines in the figure represent the collinear region between the two species.Lines of different colors represent different collinearity areas between chromosomes. The color assignment is automatically assigned by the software. For detailed drawing methods, refer to the corresponding analysis method introduction of the software(https://github.com/wyp1125/MCScanX#dual-synteny-plotter).

**A**

**
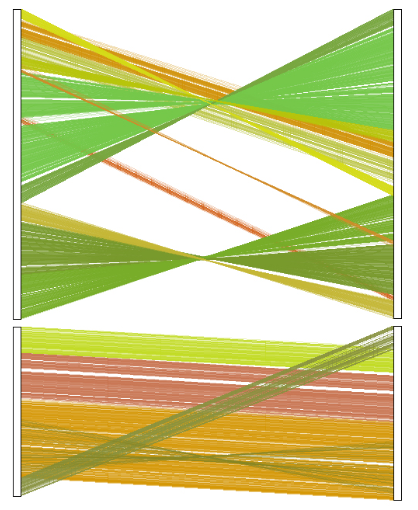
**

**Rs-P.362200 GMI1000**

**B**

**
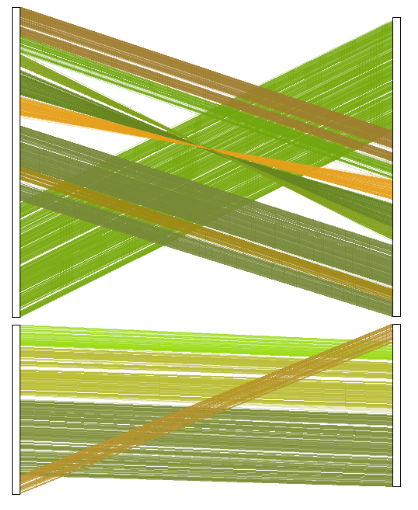
**

**Rs-P.362200 CMR15**

**C**

**
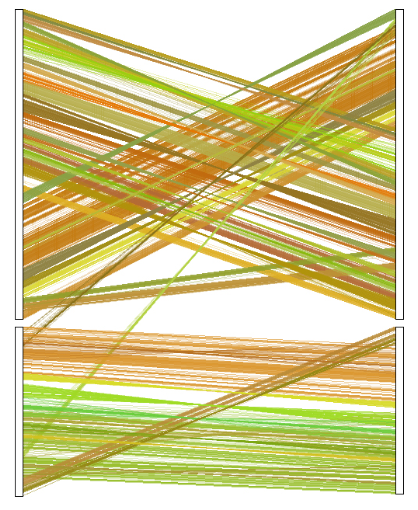
**

**Rs-P.362200 YC45**

**D**

**
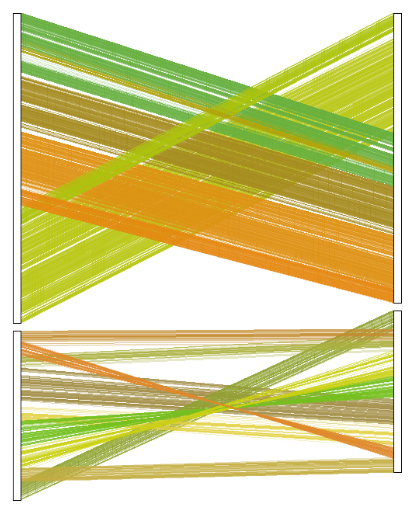
**

**Rs-P.362200 Po82**

**E**

**
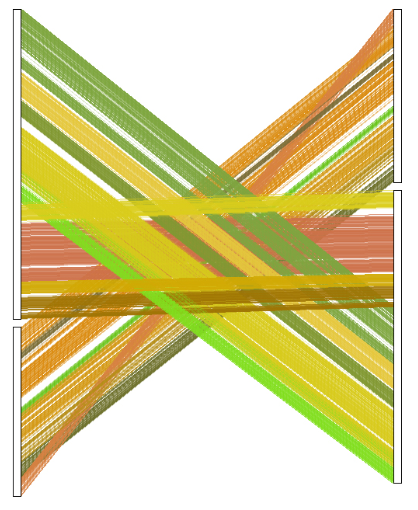
**

**Rs-P.362200 PSI07**

**F**

**
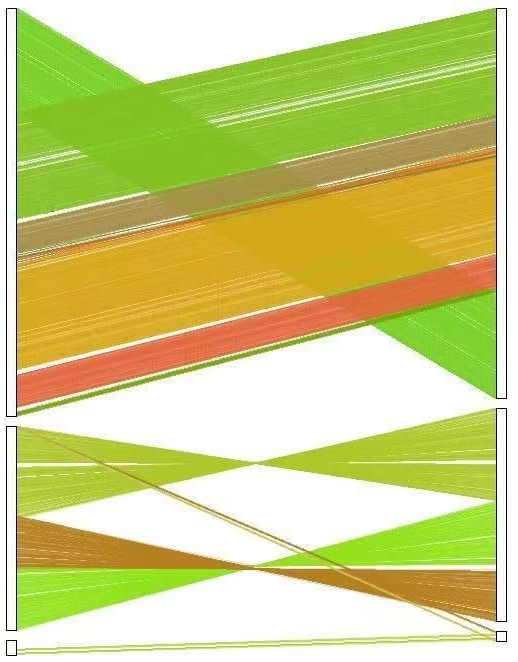
**

**Rs-P.362200 HA4-1**
