# Supplementary material for: Complete genome sequence analysis of the peanut pathogen Ralstonia solanacearum strain Rs-P.362200
Source: BMC Microbiol. 2021 Apr 19;21:118. doi: 10.1186/s12866-021-02157-7 (PMC8056632; doi:10.1186/s12866-021-02157-7)
Supplement: Supplementary file 7 — Additional file 7: Supplementary 7. Go classification statistics of specific genes of Rs-P.362200 strain. [file 12866_2021_2157_MOESM7_ESM.docx]

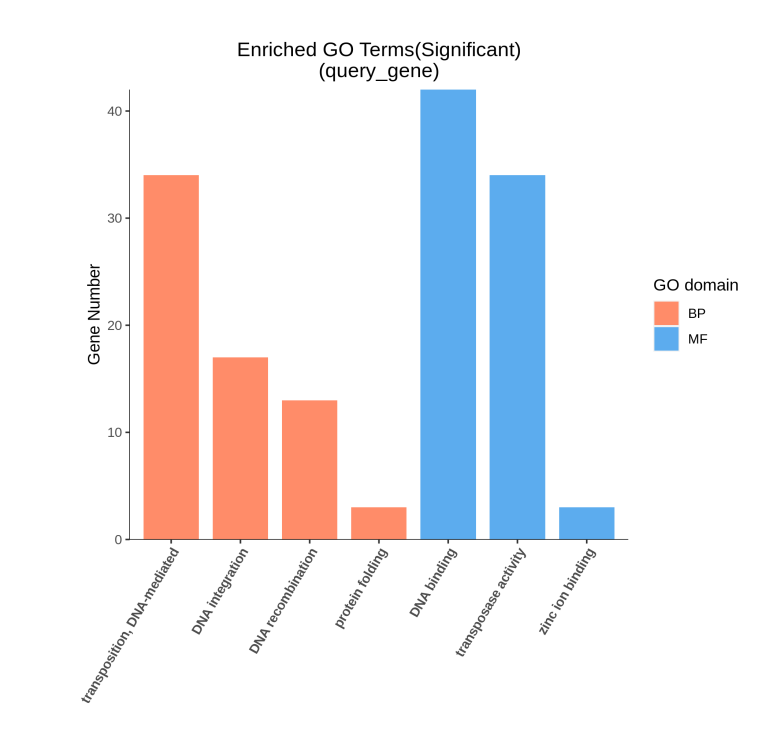


(A)


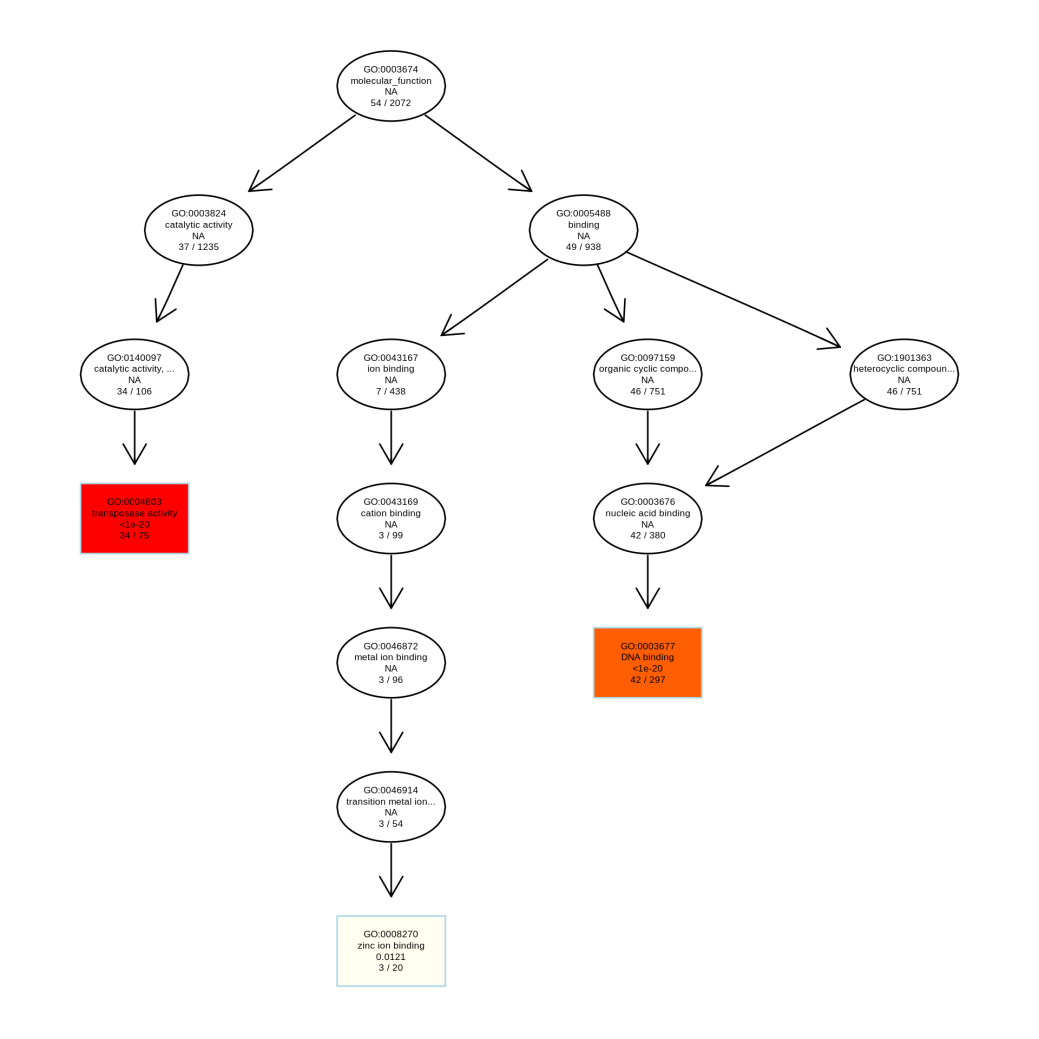


(B)


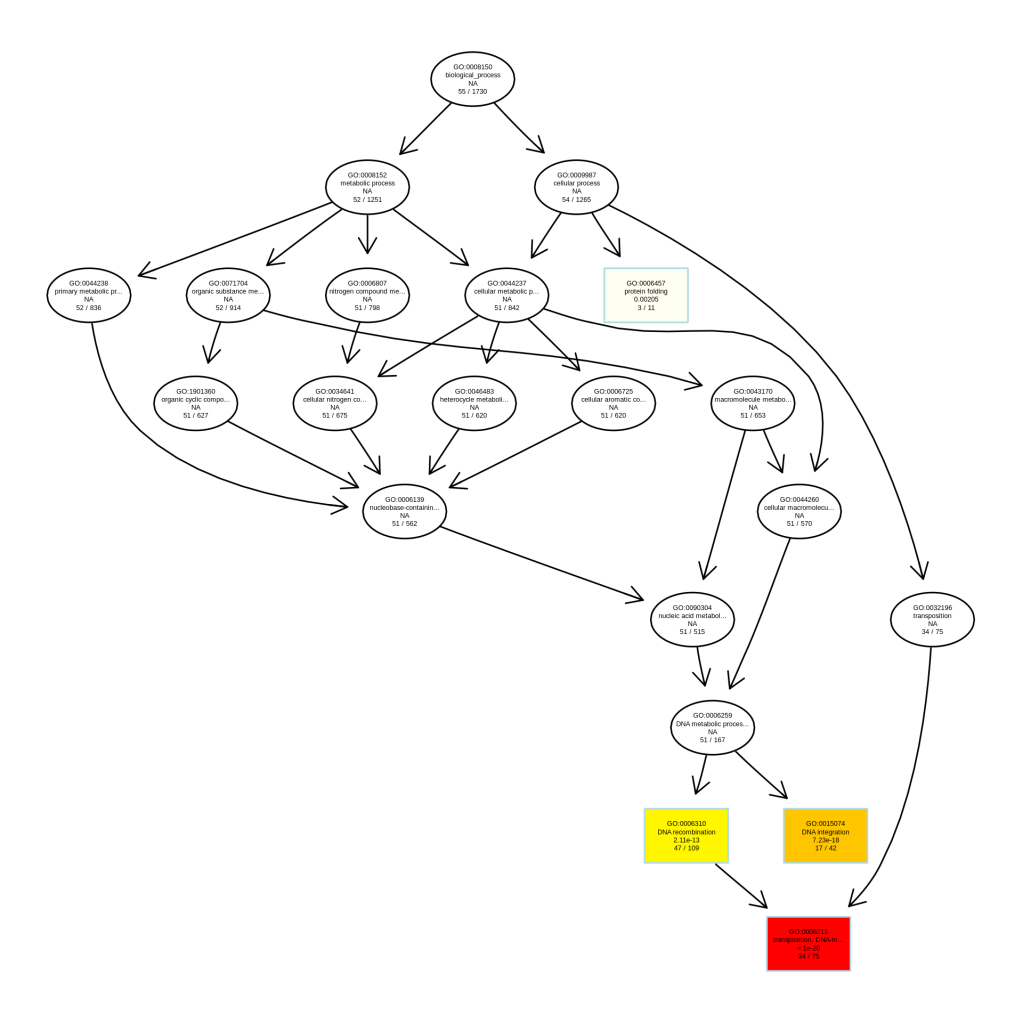


(C)


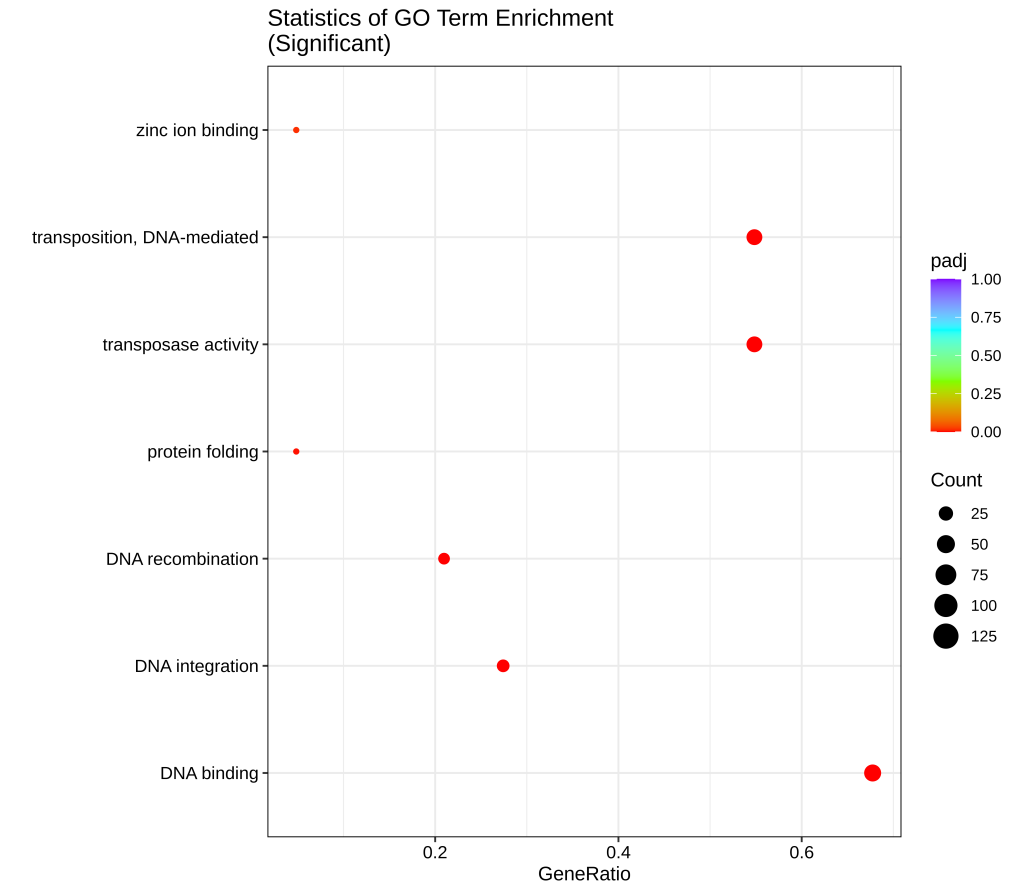


(D)

**Supplementary 7 Go classification statistics of specific genes of Rs-P.362200 strain.**

(A) GO enrichment classification. GO enrichment histogram of differential genes, the abscissa is GOterm at the next level of three categories of go, and the ordinate is the number of differential genes enriched under this term. (B)-(C) DAG graph of GO enrichment. GO directed acyclic graph (DAGgraph) can visually display the GOterm of differential gene enrichment and its hierarchical relationship. Branches represent inclusion relationships. The scope of functions defined from top to bottom becomes more and more specific. For each of the three major categories of GO (CC:cell composition, MF:molecular function, BP:biological process), the top 10 with the highest enrichment degree are taken as the main nodes of the DAGgraph, which are represented by boxes. The related GO Term is displayed together through the inclusion relationship. The depth of the color represents the degree of enrichment, and the darker the color, the higher the degree of enrichment. (D) Statistics of GO enrichment. The abscissa is GeneRatio, which indicates the percentage of the enriched genes in the annotated genes. The ordinate indicates the items on enrichment; The size of the point indicates the number of genes on enrichment, and the lower the Qvalue value indicated by color, the more significant it is.

**Note**：B and C are drawn with topGO 2.42.0 (https://bioconductor.org/packages/topGO/).
