# Supplementary material for: Complete genome sequence analysis of the peanut pathogen Ralstonia solanacearum strain Rs-P.362200
Source: BMC Microbiol. 2021 Apr 19;21:118. doi: 10.1186/s12866-021-02157-7 (PMC8056632; doi:10.1186/s12866-021-02157-7)
Supplement: Supplementary file 9 — Additional file 9: Supplementary 9. Comparative genomic analysis of R. solanacearum strains Rs-P.362200 and HA4-1. [file 12866_2021_2157_MOESM9_ESM.docx]

(A) HA4-1 Rs-P.362200


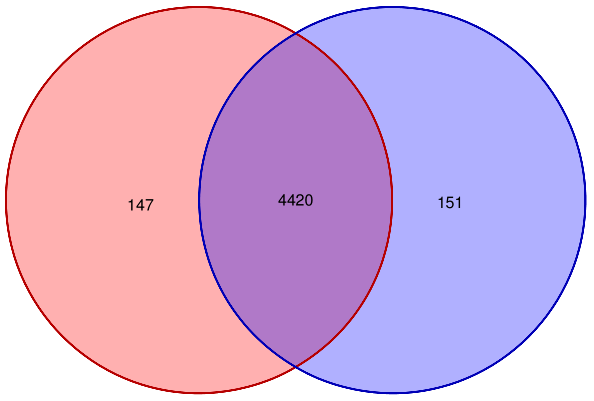

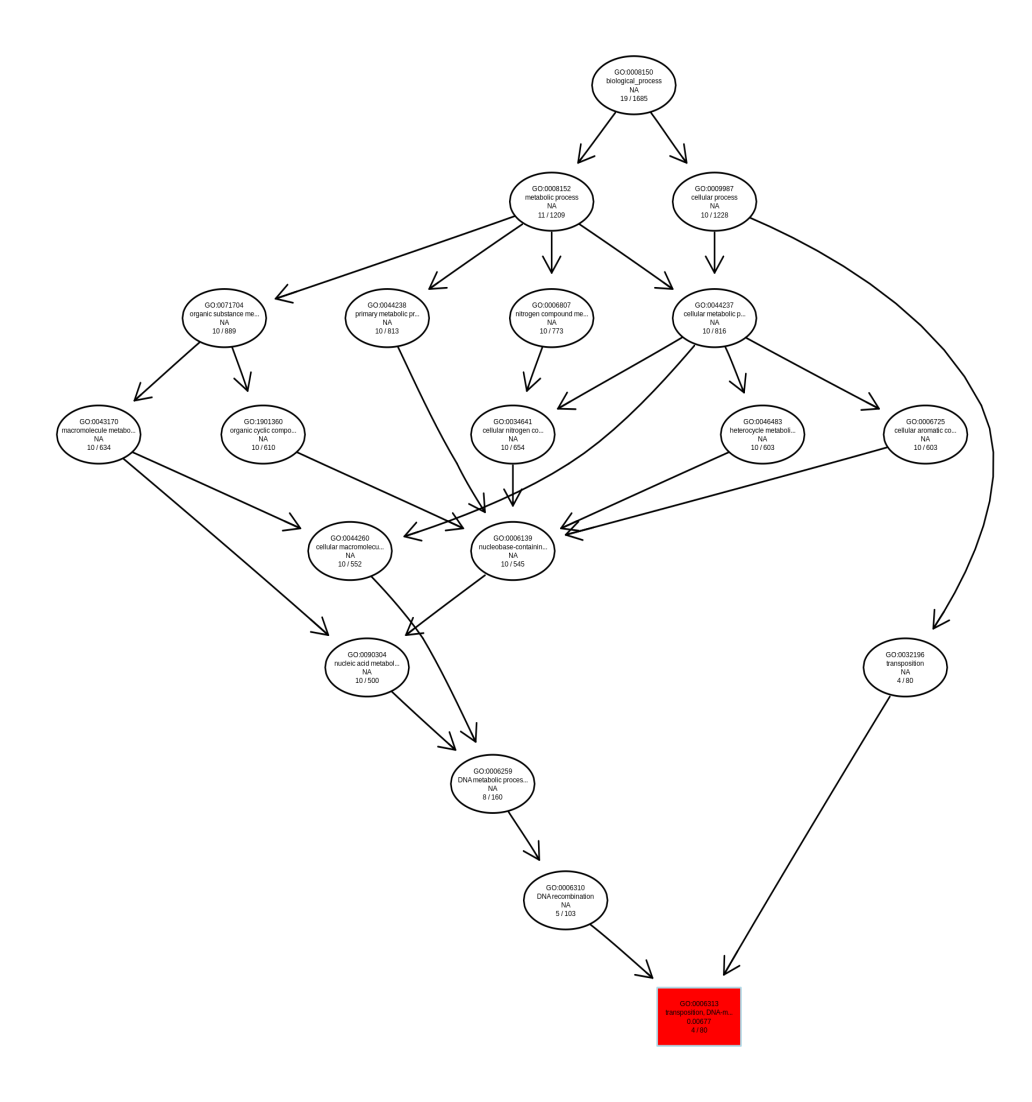


**(B)**


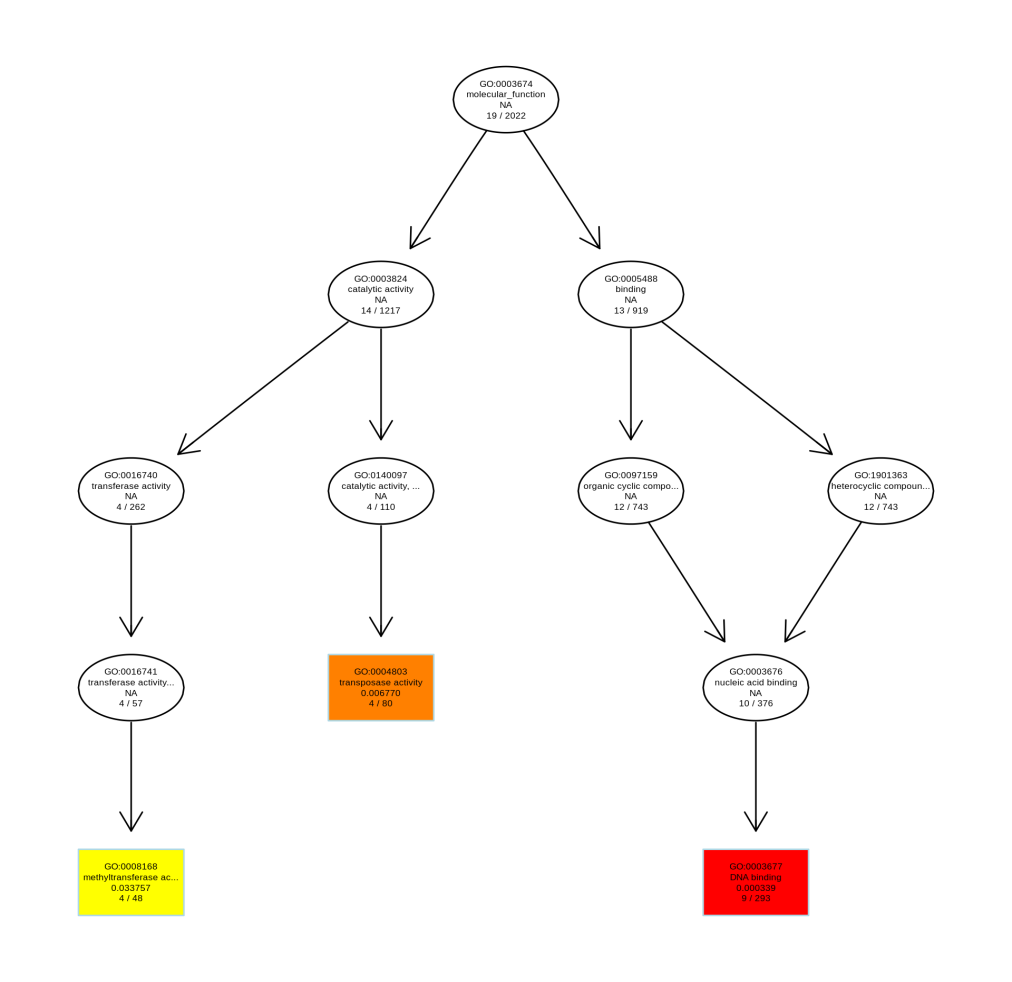


**(C)**


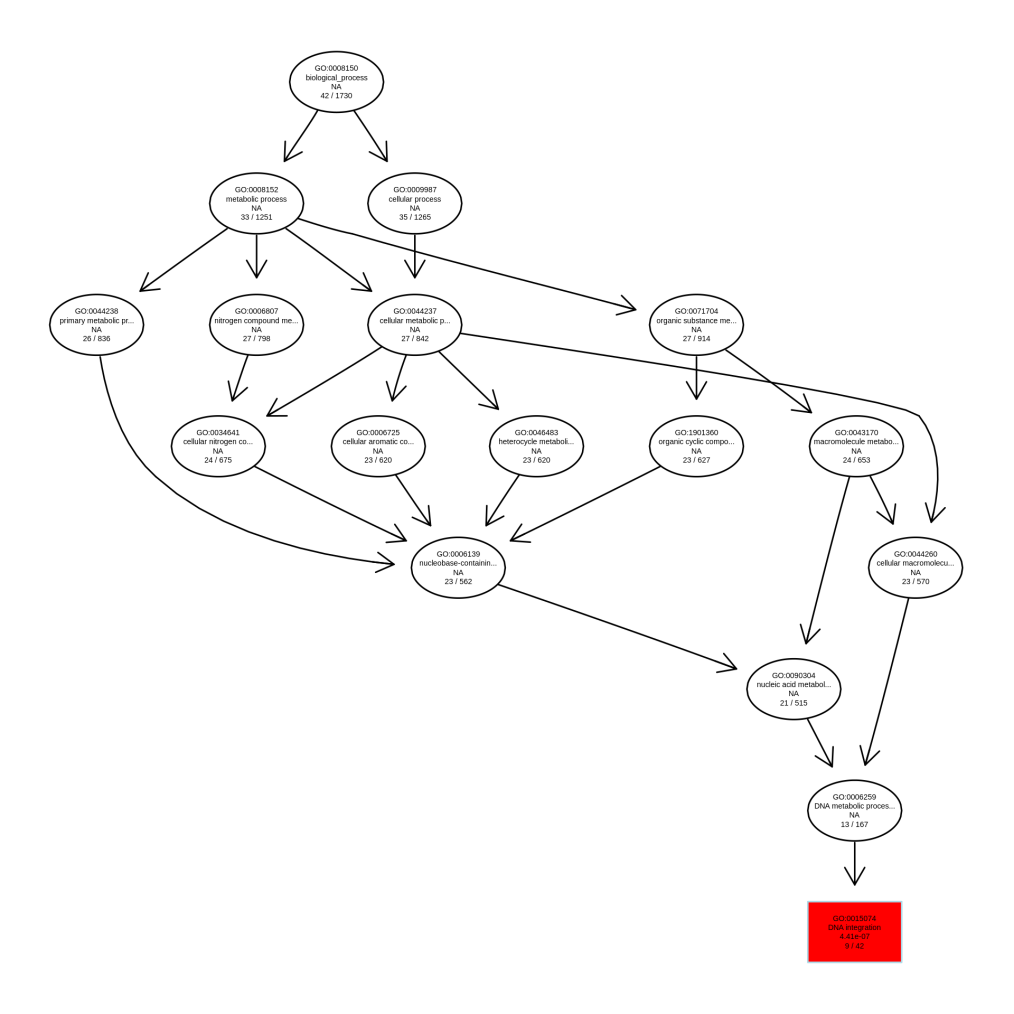


**(D)**

**Supplementary 9 Comparative genomic analysis of *R. solanacearum* strains** **Rs-P.362200 and HA4-1.** (A) Venn diagram of Rs-P.362200 and HA4-1. (B)-(C) The GO directed acyclic graph of HA4-1 specific gene enrichment. (D) The GO directed acyclic graph of Rs-P.362200 specific gene enrichment. Note: GO directed acyclic graph (DAGgraph) can visually display the GOterm of differential gene enrichment and its hierarchical relationship. Branches represent inclusion relationships. The scope of functions defined from top to bottom becomes more and more specific. For each of the three major categories of GO (CC:cell composition, MF:molecular function, BP:biological process), the top 10 with the highest enrichment degree are taken as the main nodes of the DAGgraph, which are represented by boxes. The related GO Term is displayed together through the inclusion relationship. The depth of the color represents the degree of enrichment, and the darker the color, the higher the degree of enrichment.

**Note**：B, C and D are drawn with topGO 2.42.0 (https://bioconductor.org/packages/topGO/).
